# Research on the move: exploring WhatsApp as a tool for understanding the intersections between migration, mobility, health and gender in South Africa

**DOI:** 10.1186/s12992-021-00727-y

**Published:** 2021-07-01

**Authors:** Thea de Gruchy, Jo Vearey, Calvin Opiti, Langelihle Mlotshwa, Karima Manji, Johanna Hanefeld

**Affiliations:** 1 The African Centre for Migration & Society (ACMS), University of the Witwatersrand, Johannesburg, South Africa; 2 School of Social and Political Studies, University of Edinburgh, Edinburgh, Scotland; 3 Opiti Consulting, Pretoria, South Africa; 4 Department of Geography, University of the Witwatersrand, Johannesburg, South Africa; 5 London School of Hygiene & Tropical Medicine (LSHTM), London, England

**Keywords:** Migration, Mobility, Health, Health systems, Gender, South Africa, WhatsApp, GIS

## Abstract

**Background:**

Reflecting global norms, South Africa is associated with high levels of cross-border and internal population mobility, yet migration-aware health system responses are lacking. Existing literature highlights three methodological challenges limiting the development of evidence-informed responses to migration and health: (1) lack of engagement with the process of migration; (2) exclusion of internal migrants; and (3) lack of methodologies that are able to capture ‘real-time’ data about health needs and healthcare seeking experiences over both time and place. In this paper, we reflect on a four-month pilot project which explored the use of WhatsApp Messenger - a popular mobile phone application used widely in sub-Saharan Africa – and assessed its feasibility as a research tool with migrant and mobile populations in order to inform a larger study that would address these challenges.

**Method:**

A four-month pilot was undertaken with eleven participants between October 2019 and January 2020. Using Survey Node, an online platform that allows for the automatic administration of surveys through WhatsApp, monthly surveys were administered. The GPS coordinates of participants were also obtained. Recruited through civil society partners in Gauteng, participants were over the age of 18, comfortable engaging in English, and owned WhatsApp compatible cell phones. Enrolment involved an administered survey and training participants in the study protocol. Participants received reimbursement for their travel costs and monthly cell phone data.

**Results:**

Out of a possible eighty eight survey and location responses, sixty one were received. In general, participants responded consistently to the monthly surveys and shared their location when prompted. Survey Node proved an efficient and effective way to administer surveys through WhatsApp. Location sharing via WhatsApp proved cumbersome and led to the development of a secure platform through which participants could share their location. Ethical concerns about data sharing over WhatsApp were addressed.

**Conclusions:**

The success of the pilot indicates that WhatsApp can be used as a tool for data collection with migrant and mobile populations, and has informed the finalisation of the main study. Key lessons learnt included the importance of research design and processes for participant enrolment, and ensuring that the ethical concerns associated with WhatsApp are addressed.

## Background

Recent years have seen increased attention being paid to the global governance of cross-border migration – the movement of people across international borders, including asylum seekers, refugees, migrant workers and undocumented migrants [1, 2]. Driven in many cases by high-income countries (HICs) who want to restrict the inward movement of people from low- and middle-income countries (LMICs), these discussions tend to focus on concerns around the role of the nation state and sovereignty, and push for the securitisation of cross-border migration through both border management and immigration legislation [3]. This focus on cross-border migration means that the more prevalent movements of people within their country of birth, internal migration, is often overlooked. While cross-border migrants, particularly those who are undocumented – are without the documents required to be in a country legally - face particular challenges in accessing healthcare, many of these difficulties are experienced by both cross-border and internal migrants [4, 5]. Despite attempts by global health actors to push the migration and health agenda for more than a decade in order to achieve health for all, challenges persist as cross-border and internal migrant populations continue to be left-behind in both research and policy development [6, 7]. This is particularly so for marginalised migrant groups, including those who sell sex, undocumented migrants, and people seeking asylum on the basis of their gender identity [6, 7]. Leaving migrants behind has serious implications for all, negatively affecting progress on the targets set out in the Sustainable Development Goals (SDGs) and other associated global health processes [6–10].

The South African context, associated with high levels of cross-border and internal migration, makes for an ideal case study in which to explore a mobile methodology – a methodological approach that facilitates the collection of data whilst participants migrate - for understanding the health-related experiences of migrants in ‘real-time’. South African health systems are not migration-aware nor mobility-competent, affecting access to healthcare for cross-border and internal migrants [5, 11–13]. Key populations, including sex workers and trans people, who are known to be particularly mobile, face additional barriers to access as a result of their gender and/or sexuality [14]. Furthermore, while more women are migrating into South Africa than before, they face particular challenges in the informal economy in which many work [15], accessing healthcare [16] and fulfilling care roles [17].

### Improving responses to migration and health in South Africa

Drawing on existing research literature, we highlight three specific challenges that are limiting the development of improved responses to migration and health globally, although our focus here is on South Africa: (1) the focus on individual migrants rather than on the process of migration; (2) the prioritisation of cross-border migrants and the exclusion of internal migrants; and (3) the methodological limitations for obtaining ‘real-time’ data over time and place.

Firstly, the field of migration and health tends to focus on the movement of individual migrants – including those seeking asylum – rather than on the process of migration itself [6, 9]. This often results in efforts that, for example, aim to make health systems ‘migrant-sensitive’ – often through cultural competency training for healthcare workers – rather than ‘migration-aware’, whereby systems embed the movement of people within and between countries into their design and implementation [11, 13].

Secondly, whilst limited, efforts to address migration and health focus on the movement of cross-border migrants and overlook internal migrants – those who move within their countries of birth – and intra-regional migrants, both of which are prevalent in LMIC contexts. Whilst these movements account for a significant proportion of all global migration, they remain left-behind in global migration and health governance discussions that predominantly focus on the movement of people from LMICs to HICs [9, 18, 19].

Finally, within the field of migration and health research, we have not found examples of methodologies that are able to capture ‘real-time’ data about health needs, healthcare seeking experiences, and interactions with healthcare systems over both time and place. Longitudinal research on migration and health tends to make use of repeated quantitative measures collated in a single geographic location that rely on participant recall of experiences, time, and place, such as through Demographic Health Surveillance Surveys (DHSS) [20]. Qualitative approaches for exploring migration and health over time, on the other hand, largely rely on face-to-face interactions between the researcher(s) and participants at different moments. Sometimes mobile technology – like WhatsApp – is used by researchers to remain in contact with participants, conduct interviews or, increasingly, continue ongoing participatory research [21]. However, these approaches are rarely designed to capture experiences over time and place as they are happening – something particularly important when working with migrant groups. Nor do they tend to explore the capabilities of technology as a data collection tool itself.

### WhatsApp as a research tool for exploring migration, mobility, health and gender in South Africa

In order to explore intersections between migration, mobility and the South African health system, and taking into consideration the three concerns raised above, the Migration, Gender and Health Systems (MiGHS) project - a collaboration between the Universities of the Witwatersrand (Wits) and Cape Town (UCT), the London School of Hygiene and Tropical Medicine (LSHTM) and the South African National Department of Health (NDoH) - aims to explore the use of mobile technology as a tool for researching the health-related experiences of cross-border and internal migrants in ‘real-time’. Funded by the UK MRC Wellcome Trust Health Systems Research Initiative, the project draws on previous research in the field of migration and health undertaken by the collaborators [5, 22, 23].

Following a review of the literature, we chose to explore the use of WhatsApp Messenger - a social media platform operated on smart phones – in this research. Four key reasons explain our decision to use WhatsApp: its prevalence as a means of communication in Southern Africa [24, 25]; the application’s affordability; its ease of use, including allowing the participant to respond to survey questions and share their location within WhatsApp; and – perhaps most importantly – the ability to retain contact with participants should they cross international borders [26–31].

To date, the use of WhatsApp as a tool for data collection in health research has been limited [32]. Our scoping review indicates that WhatsApp has either been used to disseminate survey tools, usually through sharing a link to an online SurveyMonkey or Google Form, or, through the creation of a WhatsApp Group as an intervention to facilitate communication between healthcare workers. We found that while some attention has been paid to the use of WhatsApp in mHealth and telemedicine, and amongst healthcare workers [33–35], there is little published that assesses the use of WhatsApp as a data collection tool in health research and communication interventions. Importantly, even within the literature that does exist, limited attention has been paid to the ethical implications of using WhatsApp or the ways in which existing social and economic inequities may affect its use.

### A pilot study

In this paper we reflect on the results of a pilot study, conducted by MiGHS, that sought to investigate the potential use of WhatsApp for a larger study the project hopes to undertake. The larger study aims to retain contact with 400 mobile study participants to be recruited in four areas of South Africa for a twelve-month period, to document their experiences of health systems usage in real-time. To explore the feasibility of this study, a four-month pilot project, on which this paper reflects, was undertaken between October 2019 and January 2020 to explore the opportunities and challenges associated with the use of WhatsApp as a research tool. This paper reflects on the successes and challenges of the pilot and how it has informed the main study. This paper does not reflect on the data collected during the pilot as the pilot sample was small and the focus of the pilot was on the feasibility and practicalities of using WhatsApp as a research tool. Nor does the paper speak to the broader interest of the MiGHS project in terms of the intersection of gender with migration and access to health systems, as this was beyond the scope of the pilot study. However, through documenting our use of the application in this research and the ways the pilot has shaped our thinking as we move into the main project, we hope to contribute insights to existing gaps in the literature on migration and health, and to the development of an emerging best practice around the use of WhatsApp in health research.

## Methods

In developing the pilot and thinking through the logistics of using WhatsApp, four key questions animated our discussions. The first was how to administer a survey through WhatsApp - being able to automate questions and responses, being able to send the survey out en masse, and ensuring that anonymity could be assured were all pivotal. The second question pertained to how we could and should ask participants to share their locations with us. The third question was how to ensure, through the recruitment process, that participants felt sufficiently comfortable with and invested in the project that they would respond to survey and location prompts across a four-month period. The final question was how to ensure that at each step of the process the privacy of participants and their information was protected.

### Data collection and management

Data was collected in three ways in the pilot:

1. A survey to ascertain the socio-demographic profile, health seeking behaviour history and migratory history of the participant was administered at the point of recruitment;
2. A monthly survey administered through WhatsApp to explore its potential for collecting and tracking health seeking behaviour across the four months; and
3. A monthly prompt for the participant to share their location with us using the WhatsApp location sharing function to generate data that would indicate if and how mobility intersects with healthcare access.

A search of available software through which monthly surveys could be administered indicated that there are very few options. The platform chosen for this project was Survey Node.^1^ Although Survey Node appears to have been primarily used in customer service evaluations, the software’s ability to interface with WhatsApp – through WhatsApp Business - and the dearth of alternatives were key factors in our choice. As such, after the successful enrolment of the participants, the WhatsApp monthly survey was sent out to each participant four times between October 2019 and January 2020. Table 1 outlines the schedule for the pilot – this was developed so that survey and location prompts were sent in a systematic manner that was clear to the whole research team. Given time constraints, surveys were administered slightly more frequently than once a month. However, they were spaced out as much as possible and in such a way so as to ensure that surveys were not sent on or too close to Christmas and New Year, but we could still gauge whether participants would respond to surveys administered at this time of year. In addition, participants were asked to share their location once a month using WhatsApp’s ‘send location’ function. Both the invitation to respond to the survey and the location sharing took place on a WhatsApp account that was run by the project team. The survey itself was administered by Survey Node using WhatsApp Business.

**Table 1:** Pilot schedule

Group 1			Group 2		
Day from enrolment	Date	Research schedule	Day from enrolment	Date	Research schedule
1	15-Oct 16-Oct	Recruit, enroll, administer enrolment survey	1	31-Oct	Recruit, enroll, administer enrolment survey
6	21-Oct	Sent out WhatsApp initiation survey via a WhatsApp broadcast	5	04-Nov	Sent out WhatsApp initiation survey via a WhatsApp broadcast
7	22-Oct	Followed up via broadcast	7	06-Nov	Followed up via broadcast
8	23-Oct	Followed up via broadcast	9	08-Nov	Followed up individually
10	25-Oct	Followed up individually	12	11-Nov	Sent data/airtime to participants who had responded to the WhatsApp initiation survey Sent out WhatsApp monthly survey 1/4
13	28-Oct	Sent data/airtime to participants who had responded to the WhatsApp initiation survey Sent out WhatsApp monthly survey 1/4 & asked for pin	14	13-Nov	Followed up individually with those who did not respond the survey Followed up with those who did not respond to the WhatsApp initiation survey
15	30-Oct	Followed up individually with those who did not respond to the survey or send their pin Followed up with those who did not respond to the WhatsApp initiation survey	26	25-Nov	Asked for location pin
39	22-Nov	Sent data/airtime to those who responded to WhatsApp monthly survey 1/4 Sent out WhatsApp monthly survey 2/4 & asked for pin	37	06-Dec	Sent data/airtime to those who responded to WhatsApp monthly survey 1/4 Sent out WhatsApp monthly survey 2/4
42	25-Nov	Followed up individually with those who did not respond to the survey or send their pin	40	09-Dec	Followed up individually with those who did not respond the survey
63	16-Dec	Sent data/airtime to those who responded to WhatsApp monthly survey 2/4 Sent out WhatsApp monthly survey 3/4 & asked for pin	49	18-Dec	Asked for location pin
65	18-Dec	Followed up individually with those who did not respond to the survey or send their pin	68	06-Jan	Sent data/airtime to those who responded to WhatsApp monthly survey 2/4 Sent out WhatsApp monthly survey 3/4
91	13-Jan	Sent data/airtime to those who responded to WhatsApp monthly survey 3/4 Sent out WhatsApp monthly survey 4/4 & asked for pin	70	08-Jan	Followed up individually with those who did not respond the survey
93	15-Jan	Followed up individually with those who did not respond to the survey or send their pin	79	17-Jan	Asked for location pin
94	16-Jan	Thank you message to all participants	89	27-Jan	Sent data/airtime to those who responded to WhatsApp monthly survey 3/4 Sent out WhatsApp monthly survey 4/4
			91	29-Jan	Followed up individually with those who did not respond the survey Asked for location to be shared via new location sharing platform
			92	30-Jan	Thank you message to all participants

Data collected through the surveys was exported from Survey Node (by a .csv file) into Excel, cleaned, and then imported into SPSS.

### The enrolment process

Our sampling was respondent driven to ensure the adequate inclusion of migrant and mobile populations [37, 38]. In the main study, we plan to enrol participants across four provinces and at healthcare facilities, as well as through organisations that work with migrant and mobile populations in order to ensure that the sample is diverse in terms of gender and migration history. For the pilot, given that we only intended to recruit ten participants, and that the primary objective was to see if and how participants responded to WhatsApp as a research tool, we recruited exclusively in Gauteng – in the cities of Johannesburg and Tshwane – through non-governmental organisations (NGOs) with whom the researchers had worked before.

Recruitment took place at three NGOs, all of whom work with migrant and mobile populations. The organisations approached potential participants, made them aware of the project, and invited them to meet with the researchers. Organisations were asked to ensure that potential participants were over the age of eighteen, could communicate in English, and owned a cell phone that was WhatsApp compatible. While we hope to include additional languages in the main study, the pilot was exclusively conducted in English. Participants were asked what their home language was and how comfortable they felt proceeding in English during enrolment. All indicated that they were happy to participate in English.

At the meeting with the researchers, potential participants were given an overview of the project, potential risks associated with involvement – these were primarily in relation to sharing data over WhatsApp – and the time commitment that it would involve. If individuals were interested in taking part, they were then recruited and started the enrolment process.

Sixteen participants were recruited into two groups – Group 1 included those recruited at the first two organisations, while Group 2 included those recruited at the third as this happened 2 weeks after Group 1 was recruited. Surveys were sent to each group according to a different schedule due to the time difference between recruitment (Table 1). In addition to administering the enrolment survey at the point of enrolment, much time was spent familiarising participants with the particularities of responding to the survey and location prompts on WhatsApp, for which we developed several practice tools (see Table 2).

**Table 2:** Research instruments

Name	Aim	When is it administered	What does it comprise of	Technological component
**WhatsApp practice surveys 1 & 2**	To get the participant comfortable with the WhatsApp/ Survey Node interface	At the point of recruitment	3 questions	In person Survey Node - WhatsApp
**Enrolment survey**	To ascertain socio-demographic profile; health seeking behaviour history; and migratory history of the participant	At the point of recruitment	66 questions - some closed, others open ended	In person Voice recorded Paper survey
**WhatsApp initiation survey**	To ascertain whether the participant is comfortable to respond without assistance to the survey and confirm enrolment	1–2 weeks after recruitment	5 questions	Survey Node - WhatsApp
**WhatsApp monthly survey**	To collect and track health seeking behaviour and mobility	Once a month for 4 months	17 questions Open and closed	Survey Node - WhatsApp

During enrolment, each participant was allocated a unique identifier and their cell phone number and service provider was recorded. All efforts were made to ensure that the participant’s contact information was correctly recorded so that they would receive the monthly surveys and data, and care was taken to ensure that this information remained confidential during and after the project. The participant’s cell phone number was added to a project cell phone and the participant was asked to save the project cell phone number on their phone. The participant was then sent the link to the WhatsApp practice survey(s). Following initial recruitments, researchers decided to develop a second WhatsApp practice survey as the researchers felt that the first was done under heavy supervision by the researcher, and would thus not necessarily indicate comfort or ability to respond to the survey. Each of the practice surveys consisted of three neutral questions including, for example, asking the participant which mobile network they used as a multiple-choice question and how they recharged their phone with data as a free text question. These surveys were designed to ensure that participants became familiar and comfortable with responding to automated questions sent through WhatsApp.

Participants were also asked to practice sending their pin locations during enrolment. After the first round of recruitment, it became clear that researchers would need to practice this with participants at the point of recruitment and ensure, for example, that location settings were turned on in WhatsApp. It was made clear to participants prior to recruitment that no real-time monitoring of their location would be undertaken. Instead, patterns in mobility over the study period and across the group of participants as a whole were what was of interest to the researchers. In addition, assurances were made that no location data would be shared with any law or immigration enforcement of any kind, and data presented in publications or at conferences would be anonymised.

During the project itself, Group 1 was asked to share their location at the same time that they were asked to respond to the monthly survey, while Group 2 was asked to share theirs ten to fourteen days after they had been asked to respond to the survey. This was done to get a sense of whether one worked better than the other, but the pilot data has not suggested any tangible difference.

Three participants were not carrying their WhatsApp compatible phones with them at the time that they were recruited; this is not uncommon in South Africa as the risk of cell phone theft is high, particularly for those reliant on public transport. Although steps were taken by researchers to ensure that these participants could complete their enrolment once they got home, the enrolment of these participants was not successful.

The final part of the enrolment process involved giving the participants approximately 50 ZAR^2^ worth of data to reimburse them for the use of their phones during recruitment. In addition to reimbursing them for any travel expenses incurred as a result of their participation in the project. A week after their recruitment, a WhatsApp initiation survey was sent to each participant. Their completion of this survey marked their successful enrolment in the pilot.

**Fig. 1:**
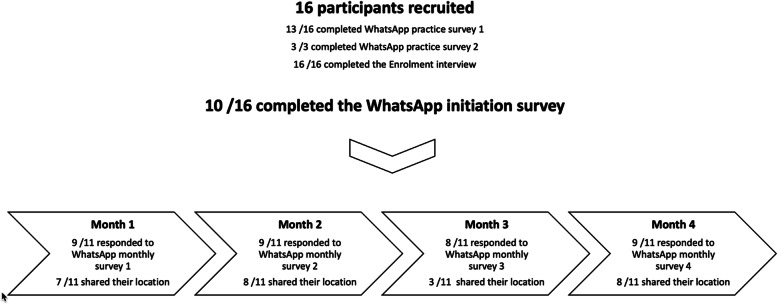
Overview of participation

Figure 1 outlines this process and the number of participants who successfully completed each step. Although ten participants completed the initiation survey, upon following up with the six participants who did not, an eleventh participant was found to be willing and able to be included in the groups receiving the monthly surveys moving forward.

### Ethical clearance

Research ethics clearance was obtained from Wits Research Ethics Committee (REC) (non-medical) (certificate H19/09/51) and the LSHTM REC (reference 17,889–1) for this study. At the point of enrolment, the project was explained to the group of potential participants as a whole. Individual participants were then approached by a single researcher and asked if they were willing to continue with the enrolment process. The details of the project – as contained in the information sheet – were then discussed, as was the informed consent form. If verbal informed consent was given this was recorded by the researcher and the enrolment process began.

## Results

As the focus of this pilot project was on the logistics and methods, and only eleven participants participated across the four-month period, this section exclusively discusses the findings with regard to the feasibility of using WhatsApp as a tool for data collection moving forward. Our analysis of these findings has led us to (a) feel cautiously optimistic about the feasibility of the main study and WhatsApp as a tool for data collection, and (b) implement appropriate changes for the main study.

### Overview of participation

As indicated above, potential participants were given an overview of the project and the opportunity to ask questions prior to choosing whether or not they would like to participate in the study. Following this information session at the first NGO, several participants left due to concerns about the amount of time it would take for them to enrol in the study as there were only three researchers present to undertake the enrolment process. No concerns were raised about the use of WhatsApp nor location sharing by participants. In addition, lacking a valid visa or permit did not appear to be a deterrent for participants, in fact many used the opportunity to express their frustrations with the documentation system in South Africa. This is in line with previous studies that have indicated that undocumented migrants are willing to report their status, so long as confidentiality and anonymity are guaranteed [5].

Of great importance to this study was establishing whether participants would continue to respond to surveys administered through WhatsApp over a period of several months. What the pilot was able to establish was that although not all 16 recruited participants responded to the survey and location tracking prompts, if participants responded to the WhatsApp initiation survey – a third practice survey sent out a week after the participants were recruited to complete their enrolment – there was a high chance that they would continue to engage with the project over the coming months. While they may not have responded to each prompt, the ten participants who responded to the WhatsApp initiation survey remained engaged over the full period of the pilot.

Figure 1 indicates the number of responses to each survey and location prompt. The low number of responses (three out of a possible eleven) to the prompt for location three are something of an anomaly, which we believe is a result of the timing of the prompt. Group 1 was asked to share their third location pin on the 16 December, a public holiday shortly before Christmas. The lack of responses from this group to this prompt account for the much smaller number of responses here and highlights the importance of taking national holidays into account when planning the main study.

Although small, the sample for the pilot included both internal and cross-border migrants; men and women; and individuals with a range of healthcare seeking histories and varied legal statuses, based on the documentation they reported holding at the time of enrolment. However, the pilot was not designed to ascertain whether there were any differences in either the engagement of participants with the research or their responses to the surveys that can be attributed to migration status, nationality or gender.

### Participant recruitment

There were three key findings with regards to the recruitment process. The first was that the enrolment. I interview allowed us to build a socio-demographic profile of the participants. While analysis of this data means very little given the small numbers recruited into the pilot, the data collected indicates that participants were able to understand and respond to the surveys administered via WhatsApp. Small changes have been made to the protocols for the main study, the rephrasing of questions for example, as informed by the pilot. Changes include adding a question about contraception to ensure that access to contraception and access to chronic medication are not conflated, and asking participants twice – once at the beginning and once at the end of the survey – whether they are on chronic medication to ensure that ambiguities around defining chronic medication were addressed.

The second finding was that the recruitment process as a whole was fairly successful in positioning participants to be able to respond to the survey and location prompts. Participants were asked how they felt and whether they had any concerns during and following the practice surveys, as well as at the end of the enrolment process. Although six recruited participants did not complete the enrolment process, this was not surprising given that three of these six participants did not have WhatsApp compatible phones with them at the point of recruitment. Moving into the main study, only participants who have their WhatsApp compatible phone with them at the time of recruitment will be enrolled.

The third finding and decision with regards to the recruitment of participants was to move the recruitment process to REDCap.^3^ The use of multiple documents during enrolment proved cumbersome as did the additional step of needing to manually enter the data into an Excel spreadsheet. REDCap is a secure web-based platform used by many in the School of Public Health at Wits (as well as across other institutions) to build, administer, and manage online databases and surveys. REDCap also has a mobile application that can be downloaded onto mobile devices. Mobile devices can then be used during the recruitment process. This will not only make the collection of data easier, but also improve the management of the database that will be created through the enrolment survey. Data collected through REDCap can be downloaded into various file formats, including .csv, which can then be imported into Excel and SPSS.

As such, the information sheet, informed consent form, checklist for practice surveys and location sharing, and enrolment survey will all be administered through REDCap. Hard copies of the information sheet will also be given to participants to ensure compliance with ethical clearance received.

### Monthly surveys

One of the key questions the pilot sought to answer was how best to conduct the monthly surveys. As indicated, we choose Survey Node as the platform on which we would build our surveys. However, although the Survey Node website talked about being WhatsApp compatible, it took some time and contact with the platform’s customer service in order to ascertain exactly how Survey Node could be used in conjunction with WhatsApp. We had imagined being able to save participants’ cell phone numbers attached to a unique identifier in Survey Node, and then send participants the survey from Survey Node directly to WhatsApp. However, survey links can only be sent via text message from the platform. Once the participant clicks on the survey link, indicating consent, they are rerouted to a WhatsApp chat with a United Kingdom number associated with Survey Node.^4^ This is in order to comply with Facebook’s consent policy. As such, the only way to send the survey to WhatsApp directly, given that we would already have informed consent from participants, is to copy and paste the survey link into a WhatsApp message, and broadcast that to participants from a project cell phone. This was the only way to circumvent a text message resembling spam as the starting point for each monthly survey. Additionally, even if numbers are saved to a unique identifier, survey responses are not linked to the unique identifier in the Survey Node database. They are exclusively linked to the number from which the responses originate. Once these limitations were clear, we were able to successfully work around them and use the platform throughout the pilot.

Over the course of the pilot, participants were each asked to respond to the monthly survey four times, and we received a total of thirty five responses to the survey (out of a possible forty four). Across these responses, nineteen instances of participants seeking healthcare were reported, of which eight were associated with some kind of challenge during this process. Challenges reported included being unable to access healthcare as the participant in question lacked the correct documentation, and complaints about waiting time at facilities. Given the small number of participants in the pilot, no conclusions can be draw from the responses themselves, but it does speak to the potential data that the main study will be able to collect.

Analysis of the data collected during the pilot did not indicate that participants were averse to answering particular questions nor that their answers were inconsistent. Analysis of the responses did point to some ambiguities and common misunderstandings with regards to the phrasing of the questions, which have subsequently been changed for the main study.

Some changes to the monthly survey have been made for the main study as informed by our analyses of the pilot. Importantly, where possible, questions and response options have been standardised across the enrolment survey and WhatsApp monthly survey by, for example, ensuring that multiple choice options are identical. Additional choices were also added to the multiple-choice questions, as informed by responses in the pilot when participants chose ‘other’ when responding to a multiple-choice question and were then given the option to explain in free text. For example, ‘physical pain’, ‘referral’, ‘contraception’ and ‘malaria’ were all added as options for the question ‘why did you seek care?’

### Location tracking

The use of WhatsApp’s location sharing function to track the location of participants proved unsustainable. Management of this data involved having to enter the WhatsApp chat between the participant and the project team, open the shared location pin, copy the data and paste it into an Excel spreadsheet that could then be imported into GIS software. While this was manageable during the pilot study given the small number of participants, a different mechanism for logging locations has been created for the main study. A secure and user-friendly web-based platform has been developed through which participants can log their location. Locations are attached to the participant’s cell phone number and automatically added to a database that can then be managed, imported into GIS software and analysed. Importantly, this platform only captures the participant’s location at the moment that they enter their cell phone number into the platform. It does not continue to track their location. This platform was tested with Group 2 the fourth and final time that they were asked to log their location, and elicited as many responses as previous prompts to send their location via WhatsApp.

While all of the location data received was from within South Africa, location data was captured from those who moved internally over Christmas and data correlated to participants reported movements as captured by the survey.

### Cell phone data

Finally, the biggest logistical concern – and something the pilot set out to explore - related to reimbursing participants for the use of their own cell phones and mobile data during the study. Three approaches were tested at the point of recruitment and during the pilot.

Initially, we had imagined that at the point of recruitment, data could be transferred from a project phone to the participant’s phone. However, we discovered that data can only be transferred between phones on the same service provider network, as such this was not a practical solution as there are four major networks in South Africa (MTN, Vodacom, Telkom, Cell C) and the researcher only has access to a project phone on a single network at the point of enrolment. To address this, on the first day of recruitment, participants were given 50 ZAR cash (sufficient to purchase, on average, 500 MB of mobile data [41]) to purchase data for a network of their choice. On the second day, participants were asked which network they used, and a member of the research team went and purchased vouchers for data that were then distributed to the participant. During the second round of recruitment, one of the researchers used her personal banking app to send data directly to each participant’s phone.

In addition, every month, prior to sending participants the survey, participants were sent 50 ZAR of data directly to their phones to enable them to respond to the survey. Understanding how we could send participants cell phone data every month has proven challenging. We had hoped to send data through Survey Node, but the pilot indicates that this is not possible. The only solution was the use of a personal bank account by one of the researchers to send data to each participant on the day that they will receive the survey. As such, a participant received 50 ZAR worth of data and then, a few minutes later, a broadcast message on WhatsApp with a link to the survey every month.

## Discussion

Due to both the novelty of WhatsApp as a tool for data collection, in addition to the limited literature documenting its use in health research to date, each stage and element of the pilot project was subject to detailed discussion during the project’s development, its implementation and following its conclusion. Although the focus of this paper has not been on the data collected through the pilot, due to the size of our sample, an analysis of the data has indicated that participants were able to understand the questions and respond to both multiple choice and free text questions appropriately. While some questions in both the enrolment and monthly survey instruments have been rephrased for the main study as informed by the pilot, the data from the pilot has indicated that we will be able to analyse the responses in the main study in order to address the research objectives of the MiGHS project as a whole.

The concerns that dominated our discussions however related to whether or not the research was ethical and was being conducted in ways that protected participants, and whether there were additional ways to streamline the mechanisms underpinning the research.

There were three key ethical considerations in this project. The first pertained to its longitudinal nature and ensuring that consent was ongoing. It was felt that through ensuring a rigorous informed consent process at the point of recruitment and the need for participants to physically ‘opt in’ every month by responding to the survey questions or logging their location, participants were in a position to decline to remain involved in the project. By continuing to opt in through responding to prompts, participants can thus be understood to be giving continued consent.

This links to the second ethical concern - the use of mobile phone numbers and anonymity. While participants’ names were never recorded, their numbers were vital for the use of Survey Node and WhatsApp. However, within the South African context, cell phone numbers are not key identifiers of individuals. Several individuals may share one cell phone number, numbers may not be registered in the users’ name and are easily discarded and replaced, and reports indicate that often individuals have more than one cell phone or cell phone number to take advantage of various offers by network providers [42]. While this may have some ramifications for our research, it means that participants are not exposed to any undue risk by sharing their cell phone numbers with us. It does mean, however, that participants’ responses could be seen by other users of a phone that is shared or that surveys could be responded to by someone who is not the enrolled participant. While this is difficult to protect against, a discussion about this at the point of enrolment is necessary to ensure that potential participants, who are best placed to understand the dynamics of their own phone use, understand these risks and make an informed decision as to whether or not they participate in the study. Whilst it is not possible to remove these risks entirely, in future research participants could be encouraged to delete their survey responses once completed or enable WhatsApp’s ‘disappearing messages’ function for the chat with Survey Node.

The third ethical consideration pertained to the sharing of location data. Ultimately, the WhatsApp share location function generated data in a format that was too cumbersome for the project team to manage. In addition, privacy concerns associated with shared, lost or stolen phones remain. To address this, we developed and used our own secure platform for location sharing towards the end of the pilot, which we will continue to use in the main study.

Finally, the biggest logistical concern related to reimbursing participants for the use of their own cell phones and mobile data during the research. During the research we tried a number of strategies including using the personal banking app of one of the researchers to transfer data to the participants, as discussed in the results. Although we were able to make do for the pilot, none of these options are realistic when we consider recruiting over 400 participants for the main study. Moving forward, it is clear that at the time of enrolment, pre-purchasing a large quantity of vouchers for each of the service providers and equipping each researcher with several vouchers from each network is the best way to proceed.

With regards to the monthly data sent to participants the only real option – which was done in the pilot and we will continue to do in the main study – is to make use of one of the researcher’s personal banking accounts to send data directly to each participant on the day that they will receive the survey. While this remains a cumbersome part of the research method, streamlining and automating this part of the process has not been possible given the constraints of university financing systems and banking systems. Ideally, this process would utilise an institutional bank account that offers the same functionality as a personal bank account.

### Limitations

While we believe our pilot study to have been sufficiently successful as to warrant optimism about the main study, several limitations with both WhatsApp as a data collection tool and our piloting of it remain.

A central limitation of this pilot is the small number of participants. While engagement by participants was fairly consistent and high, it is difficult to say with any degree of certainty whether a much larger sample will behave in a similar way. Our sample for the pilot were all recruited through NGOs and, as such, many have had a higher level of digital literacy and felt more entitled to reflect on and able to articulate their experiences accessing healthcare. In addition, due to the small size of our sample, it is unclear how existing inequities, including the way in which access to mobile devices is gendered, may affect participation in the main study. While WhatsApp is a fairly cheap way to communicate, for many in South Africa access to a WhatsApp compatible phone remains prohibitively expensive. As such, the necessity for participants to have access to a WhatsApp compatible cell phone will introduce potential biases in the sample and lead to oversampling of some demographics. However, the implications of inequities in access to WhatsApp will only become apparent in the main study.

An additional limitation as a consequence of the size of the sample is that we were not able to explore gender and the intersection of gender with migration and with access to healthcare in the pilot. However, the data does indicate that these are intersections that we will be able to explore with a larger sample in the main study.

In addition, the use of WhatsApp is itself a potential limitation: it is not a quick and easy research fix. In this case, a thorough enrolment process completed face-to-face; constant time and input from researchers to ensure that participants were receiving prompts, cell phone data and responding to questions; and access to mobile devices and consistent and reliable internet for researchers were all vital. This is in addition to the importance of finding ways to address the limitations of institutional financing systems, which requires research teams to have the ability to foot research costs upfront in order to conduct the research. While this is not meant to dissuade anyone from considering the potential of WhatsApp as a research tool, it is important to note that this approach to research is not to be undertaken lightly and without adequate preparation.

Finally, use of WhatsApp requires researchers to keep abreast of changes in the functionality and security of the application itself and developments in the regulations governing the collection and storage of private information across different contexts. In South Africa, for example, the *Protection of Personal Information Act* (POPIA) (2013) will come into effect in July 2021 [43]. The implications of this for research remain to be seen as Research Ethics Committees are still developing guidelines for research within this new legislative context.

## Conclusion

The successful completion of this pilot points to the feasibility of and potential for the use of WhatsApp in health research, particularly with migrant and mobile populations. We have shown that WhatsApp can be used as a platform for undertaking repeat surveys over time and place, without needing to redirect the participant to another platform and survey tool that may not be compatible with their device and which may lead to increased costs for the participant. However, the mechanics of research that uses WhatsApp as a research tool need to be carefully thought out. This is particularly true during the enrolment process; this is the only point at which the researcher meets with the participant face-to-face. The use of WhatsApp provides the opportunity to gather real-time data from participants over a period of time. Data that, in this case, will hopefully go some way to improving our understandings of the ways in which migrant and mobile individuals move and their health experiences and interactions with the healthcare system in South Africa. However, the use of WhatsApp can – if not managed carefully - place participants at increased risk due to both the nature of the data that is collected and the method of capturing data through the application. As with any research approach, due consideration must be given to ensure that the data provided by participants is protected and that they are not exposed to any risk through the research process. The use of mobile technology heightens the importance of the principle of do no harm.

In paying careful consideration to the use of WhatsApp, we have developed mechanisms to rigorously document our experiences of the use of WhatsApp and to detail our approach to addressing the associated ethical challenges – key gaps in the current literature as identified by our scoping review [32]. It is difficult to determine if we have adequately understood and taken into account the ways that existing inequities are reflected in the research methodology due to the limited number of participants in the pilot study [42, 44]. However, the pilot suggests that ownership of WhatsApp-compatible cell phones is sufficiently widespread to allow for this kind of a study. In addition, ensuring that participants are provided with data to enable their participation ensures that participants are not excluded due to the cost of participation.

Finally, the pilot demonstrates that the use of WhatsApp allows for research that addresses the three concerns we raised with the state of research on migration and health. Firstly, through continuous engagement with participants over both time and place a shift in focus from the experiences of individual migrants to a better understanding of how the migration process intersects with access to healthcare will be facilitated. Secondly, through respondent driven sampling, internal migrants – and, therefore, internal migration – are not left-behind. Finally, the use of WhatsApp allows for the collection of ‘real-time’ data over time and place. It suggests, through the ability to focus on the process of migration, including internal migration, and opportunities it provides to elucidate the ways in which this process intersects with access to healthcare and gender, that this kind of research method has real potential.

*The findings from this pilot study inform were used to inform the finalisation of the methodology for our main study, which has been postponed indefinitely due to the Covid19 pandemic.*

## Data Availability

All data generated or analysed for this paper are included in this published article [and its supplementary information files].

## Abbreviations

ACMS: The African Centre for Migration & Society, University of the Witwatersrand
GCM: Global Compact for Safe, Orderly and Regular Migration
GCR: Global Compact for Refugees
HIC: High-income country
LMIC: Low- and middle-income country
LSHTM: London School of Hygiene and Tropical Medicine
MiGHS: The Migration, Gender and Health Systems project
NDoH: National Department of Health
NGO: Non-governmental organisation
REC: Research Ethics Committee
SDG: Sustainable Development Goals
UCT: University of Cape Town
UHC: Universal Health Coverage
Wits: University of the Witwatersrand

## Footnotes

1 Survey Node is a trading name of ESJ Software Ltd., a UK software development company that specialises in developing and supporting innovative cloud and enterprise solutions for telecom networks, value added service providers and customer service focused organisations [36].

2 2,8 USD at the time of writing (August 2020). We say approximately as some networks do not have a data or airtime voucher that is worth exactly 50 ZAR.

3 ‘REDCap is a secure web platform for building and managing online databases and surveys. REDCap’s streamlined process for rapidly creating and designing projects offers a vast array of tools that can be tailored to virtually any data collection strategy.’ [39, 40]

4 ‘To initiate a message to a number via WhatsApp there must be an explicit confirmed opt-in obtained prior to message initiation.’ (personal communication with Survey Node, 9 October 2019)
